# Characteristics and molecular mechanism of drug-tolerant cells in cancer: a review

**DOI:** 10.3389/fonc.2023.1177466

**Published:** 2023-07-07

**Authors:** Xian-Wen Liang, Bing- Liu, Jia-Cheng Chen, Zhi Cao, Feng-ran Chu, Xiong Lin, Sheng-Zhong Wang, Jin-Cai Wu

**Affiliations:** ^1^ Department of Hepatobiliary and Pancreatic Surgery, Hainan General Hospital (Hainan Affiliated Hospital of Hainan Medical University), Haikou, China; ^2^ Department of Gastrointestinal Surgery, Central South University Xiangya School of Medicine Affiliated Haikou Hospital, Haikou, China

**Keywords:** cancer, drug tolerance, characteristics, molecular mechanisms, therapeutic strategies

## Abstract

Drug resistance in tumours has seriously hindered the therapeutic effect. Tumour drug resistance is divided into primary resistance and acquired resistance, and the recent study has found that a significant proportion of cancer cells can acquire stable drug resistance from scratch. This group of cells first enters the drug tolerance state (DT state) under drug pressure, and gradually acquires stable drug resistance through adaptive mutations in this state. Although the specific mechanisms underlying the formation of drug tolerant cells (DTCs) remain unclear, various proteins and signalling pathways have been identified as being involved in the formation of DTCs. In the current review, we summarize the characteristics, molecular mechanisms and therapeutic strategies of DTCs in detail.

## 1 Background

Every year, approximately 10 million people die from cancer across the world (1). Currently, the main treatments for cancer include surgical resection, chemotherapy, radiation therapy, immunotherapy, targeted therapy and Chinese medicine. However, each method has its own clinical limitations. Chemotherapy-based systemic treatment still plays a vital role. In particular, targeted therapy and immunotherapy, which have gradually emerged in recent years, have exhibited some efficacy in the treatment of certain tumours. Nevertheless, whether it is chemotherapy, targeted therapy or immunotherapy, there exists widespread drug resistance, which can hinder the treatment of tumours and cause disease recurrence (2). Therefore, investigating the mechanisms of tumour drug resistance development and preventing the emergence of drug-resistant cells remain major challenges for current researchers.

However, the mechanisms by which tumour drug resistance develops are complex and result from the combined action of multiple proteins and signalling pathways. Previous studies have indicated that some cells in tumours that have genetic mutations before drug treatment and have stable drug resistance, and that they survive drug pressure and become the dominant cells, eventually resulting in tumour drug resistance (3). This cellular resistance is known as primary drug resistance. Recently, an increasing number of studies have found that some cancer cells exhibit a sensitive response in the first treatment with a drug, and when treated again with that drug, resistance occurs, known as acquired drug resistance. Acquired drug-resistant cells may arise due to either epigenetic or genetic alterations, and the two are not mutually exclusive (4, 5). Actually, a high proportion of cancer cells acquire stable drug resistance from scratch (6, 7). This fraction of cells first enters a drug-tolerant persistent state (drug tolerant state) under drug stress, and in this state, they gradually acquire stable drug resistance through adaptive mutations (7, 8), which can therefore become drug tolerant cells (DTCs). After removing the drug, these DTCs quickly regain their ability to proliferate and become susceptible to the drug again after a period of time, suggesting that the DTCs have not yet acquired stable drug resistance. The concept of DTCs is borrowed from microbiology for drug-tolerant bacteria, i.e., non-growing or slow-growing bacteria that survive antibiotic treatment without developing resistance to the drug (9, 10). In the cancer field, they are defined as a subpopulation of cancer cells that survive high selective pressures of cytotoxic drugs without the development of drug resistance (11). DTCs show a lot of similarities to stable resistant cells, but the differences are also obvious, as shown in **Table 1**. DTCs are an intermediate state in the formation of stable drug-resistant cells. Therefore, blocking the formation of DTCs or targeting DTCs for treatment is expected to reduce the number of stable drug-resistant cells by lowering the formation of stable drug-resistant cells, which can thus improve the therapeutic outcome of tumours.

**Table 1:** Differences and similitudes regarding DTCs and stable drug-resistant cells.

	DTCs	Stable drug-resistant cells
Difference	Epigenetic changes, no genetic mutations (7, 12)	Gene mutations (6)
	Can be reversed into sensitive cells (12, 13)	Irreversibility (5)
	An intermediate transition state; can further develop into drug-resistant cells (12, 14)	Steady state (2, 7)
	Cell stasis (12, 14)	Proliferative activity (7, 8)
Similitude	Insensitive for drug treatment (15); Anti-apoptotic (6); Immune escape (6, 8)	

**Figure 1:**
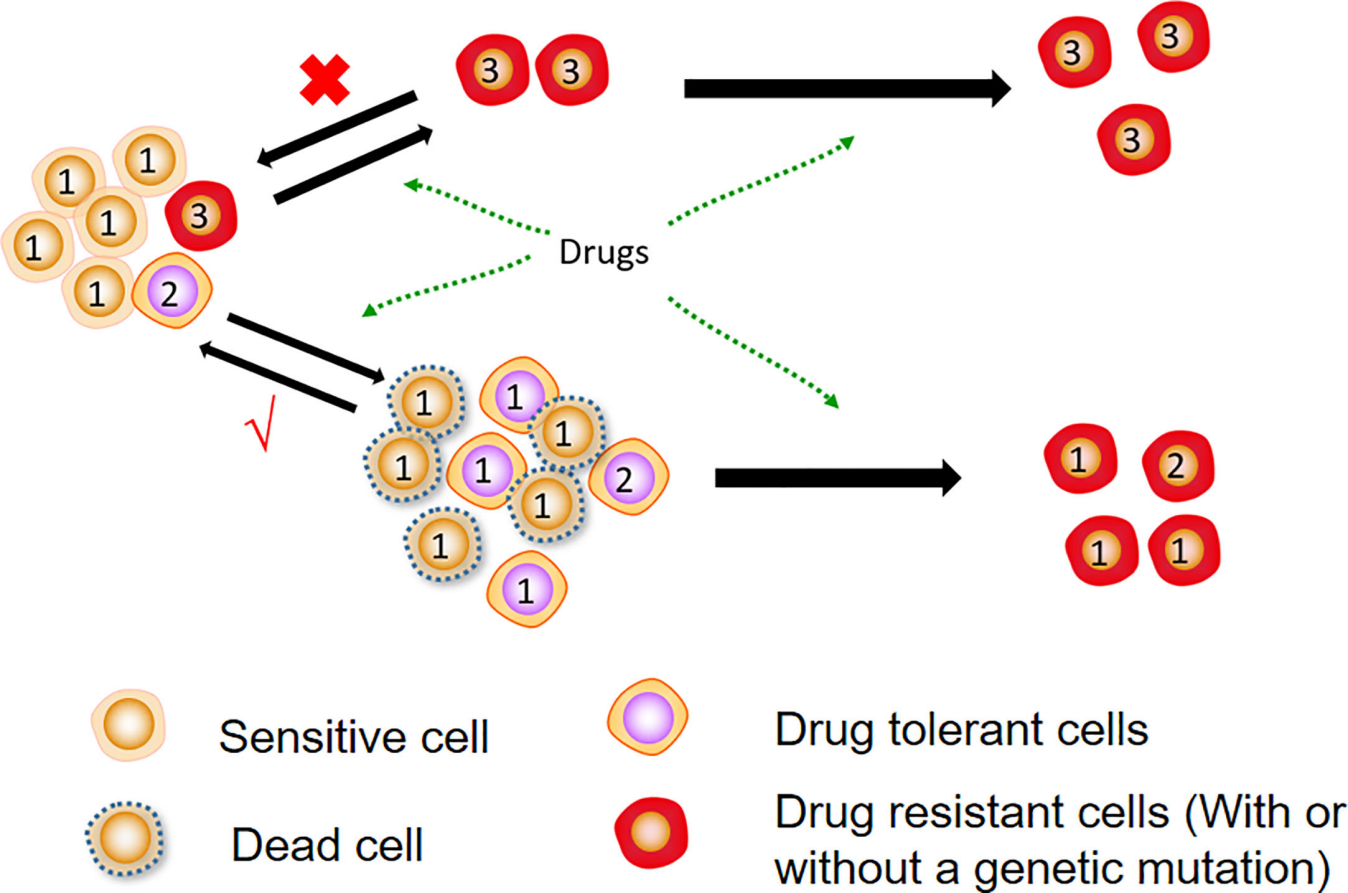
Sources of DTCs. Most DTCs are formed from sensitive cells after drug treatment and do not undergo genetic mutations. They can be reversed into sensitive cells again after drug removal. Further treatment of DTCs causes genetic mutations that form stable resistant cells.

The origin of DTCs remains unclear, with most studies showing that DTCs are produced by drug-induced overproliferation to a dormant state rather than drug selection (5, 12). Other studies reveal that DTCs are present in primitive cancer cell populations and are selected by therapeutic drugs (4, 14). *In vitro* trials have indicated that DTCs can resume proliferation after cessation of drug treatment and that their progeny remains sensitive to the initial therapeutic agent (12, 13). Furthermore, clinical evidence also demonstrates that some patients’ tumours are still effective for the same treatment after a ‘drug holiday’ from initial treatment (16). The source of DTCs is displayed in **Figure 1**.

## 2 Characteristics of DTCs

Although there is still no consensus on the origin, phenotypic characteristics and identification markers of DTCs, numerous recent studies have indicated that they share some common features. The two main characteristics of DTCs include cell proliferation arrest and reversible drug sensitivity. In addition, enhanced resistance to apoptosis and adaptive survival and a senescence-related phenotype are also vital features of DTCs, which are discussed in detail below.

### 2.1 Stagnant cell proliferation

Resting dormancy is one of the main characteristics of DTCs. Tumour cells under drug pressure temporarily enter a dormant state in which they do not proliferate and are known as DTCs (12). Regardless of the nature of the drug and the category of cancer, the cell cycle arrest phenotype observed in DTCs is a commonly seen feature (15). In DTCs, cell cycle-related gene expression and transcriptome enrichment are reduced to a great extent (17). Moreover, it has been demonstrated that targeting key regulators of the cell cycle and cell division, both *in vivo* and *in vitro*, is effective to reduce the production of DTCs in several cancer types.

Even though DTCs have dormant properties, they are not exactly the same as dormant cells, which are often present in primary tumour cells prior to drug treatment and are not dependent on drug stress (18). By contrast, DTCs are temporary dormant states entered by active proliferating cells after drug treatment, and are reversible, similar to the resting state of stem cells. In fact, the relationship between DTCs and stem cells still remains unclear, but it is certain that DTCs share many of the same properties as stem cells (19). For example, DTCs of gastric cancer express various stem cell markers, such as LGR5, TROY and ALDH1A1, and ALDH1A1 can promote the formation of DTC of acute myeloid leukemia cells through up-regulating mTOR (20).

### 2.2 Reversible drug sensitivity

The reversibility of sensitivity to therapeutic agents is another key feature of DTCs. Specifically, tumour cells that enter the DT state can be re-sensitised to the initial therapeutic agent after a period of drug removal. Sharma et al. first reported DTCs when treating epidermal growth factor receptor mutant non-small cell lung cancer PC9 cells with the tyrosine kinase inhibitor erlotinib induced the formation of DTCs after removing the drug. These DTCs could regain their proliferative capacity and were again sensitive to erlotinib after a period of time (12). By contrast, in the subsequent studies, the same phenomenon has been observed in DTCs induced in tumour cells from melanoma, breast and colon cancer (21, 22). Therefore, it is suggested that re-sensitisation after tolerance to the initial treatment is not specific to a particular tumour, but is a common feature of DTCs.

### 2.3 Increased resistance to apoptosis

Apoptosis is usually regulated by the extrinsic or intrinsic activation of protein hydrolases, which ultimately triggers programmed cell death. Currently, it is believed that resistance to apoptosis is closely associated with developing drug tolerance in tumour cells, although it is unclear whether apoptosis in DTCs is a defective form of apoptosis. As a dormant state, DTCs have significantly reduced levels of RNA transcription and translation, and their post-transcriptional modifications are also involved in maintaining the stability of DTCs (23). In DTCs obtained by treating EGFR-mutated non-small cell lung cancer cells (NSCLC) with gefitinib, a significant upregulation of the mTOR-mediated anti-apoptotic protein MCL1 could be found, indicating that DTCs are closely associated with anti-apoptosis (24). Hata et al. found that patient-derived DTCs subject to treatment with EGFR-targeted therapy for 15 days experienced an obviously lower proportion of apoptosis and reduced dependence on EGFR activation, while increased sensitivity to BCL-x and BCL2 inhibitors (8). In addition, previous studies have shown that BCL-x and BCL-2 play an important role in anti-tumour cell apoptosis (25, 26). Reticulin 4 (RTN4) is able to sequester BCL2 and BCL-x in the endoplasmic reticulum, preventing them from entering the mitochondria and therefore lowering their anti-apoptotic function (27). In breast cancer DTCs, specific missense mutations in RTN4 have been reported, which can thus protect DTCs against apoptosis through an intrinsic pathway (28). Interestingly, several studies have found that DTCs are more prone to ferroptosis. For example, Hiroto et al. found that unlike PC9 cells, oxitini-mediated DTCs derived from PC9 lung cancer cells were highly sensitive to ferroptosis inducer RSL3, indicating that DTCs were susceptible to ferroptosis (29). The study of Halime et al. also found that DTCs can increase the sensitivity to ferroptosis by inhibiting GPX4 (30). Inhibition of KDM5A increases MPC1 expression in head and neck cancer DTCs, thereby reducing susceptibility to ferroptosis (31).Several drugs targeting the apoptotic pathway in DTCs have already been developed, such as ABT263, a specific inhibitor of the anti-apoptotic proteins BCL-2, BCL-W and BCL-XL, increasing the sensitivity of DTCs to drugs (32).

### 2.4 Increased adaptive survivability

Cell plasticity refers to the ability of cells to adopt different phenotypic states transiently (33). Constant nutrient and oxygen deprivation characteristic of the chaotic tumor microenvironment could trigger the plasticity gene programs (34). Phenotypic plasticity of cancer cells in response to different microenvironments may result in drug tolerance of tumors (35).This kind of plasticity is mainly achieved through dedifferentiation, stem-like properties, epithelial-to-mesenchymal EMT (EMT)-like transitions, etc. There is a growing consensus that drug resistance in tumour cells is closely correlated with cellular plasticity (36). For example, the expression of AXL, EGFR and NGFR, which are related to cell dedifferentiation and plasticity, is significantly higher in melanoma after drug treatment (37). A significant increase in AXL expression was also detected in patients with progressive melanoma after targeted therapy (38). In addition, it has been demonstrated that AXL activation is involved in the generation and maintenance of DTCs in lung cancer treated with axitinib, and that the application of AXL inhibitors in combination with targeted drugs is effective in preventing DTCs both *in vitro* and *in vitro (* 39). Similarly, in tumour tissue from metastatic lung cancer, single cell transcriptome analysis demonstrated that some tumour cells possessed alveolar regenerative cell characteristics, suggesting that tumour cells were transforming to primitive cells when induced by drugs. In addition, it has been confirmed that EMT is a source of phenotypic and functional plasticity in tumour cells, which can thus increase the adaptability of cancer cells to drug selection pressure (40). Moreover, EMT enrichment has been found in both EGFR-mutated lung cancer and melanoma DTCs, which can further demonstrate that DTCs are plastic (8, 41).

### 2.5 Ageing-related phenotypes

Based on other related studies, it has been discovered that DTCs are closely associated with cellular senescence and that cellular senescence may be one of the mechanisms by which DTCs acquire drug resistance. Regarding senescence, it is a physiological response of cells to signals such as DNA damage, telomere shortening and overstimulation, and radiotherapy can also induce cellular senescence, i.e., therapeutic senescence. Tumour cell senescence shares some common features with normal cell senescence, including resistance to apoptosis, production of a large number of secreted factors, and formation of staining foci (42). However, senescent tumour cells can re-enter the cell cycle through modification of the tumour microenvironment and epigenetics (43), which may be one of the potential mechanisms of drug resistance in tumour cells. The upregulation of the transcriptional program associated with senescence and senescence-associated ß-galactosidase activity has been observed in DTCs from various tumours, while downregulation occurs after drug withdrawal (44, 45).

## 3 Main molecular mechanisms of DTCs formation

**Figure 2:**
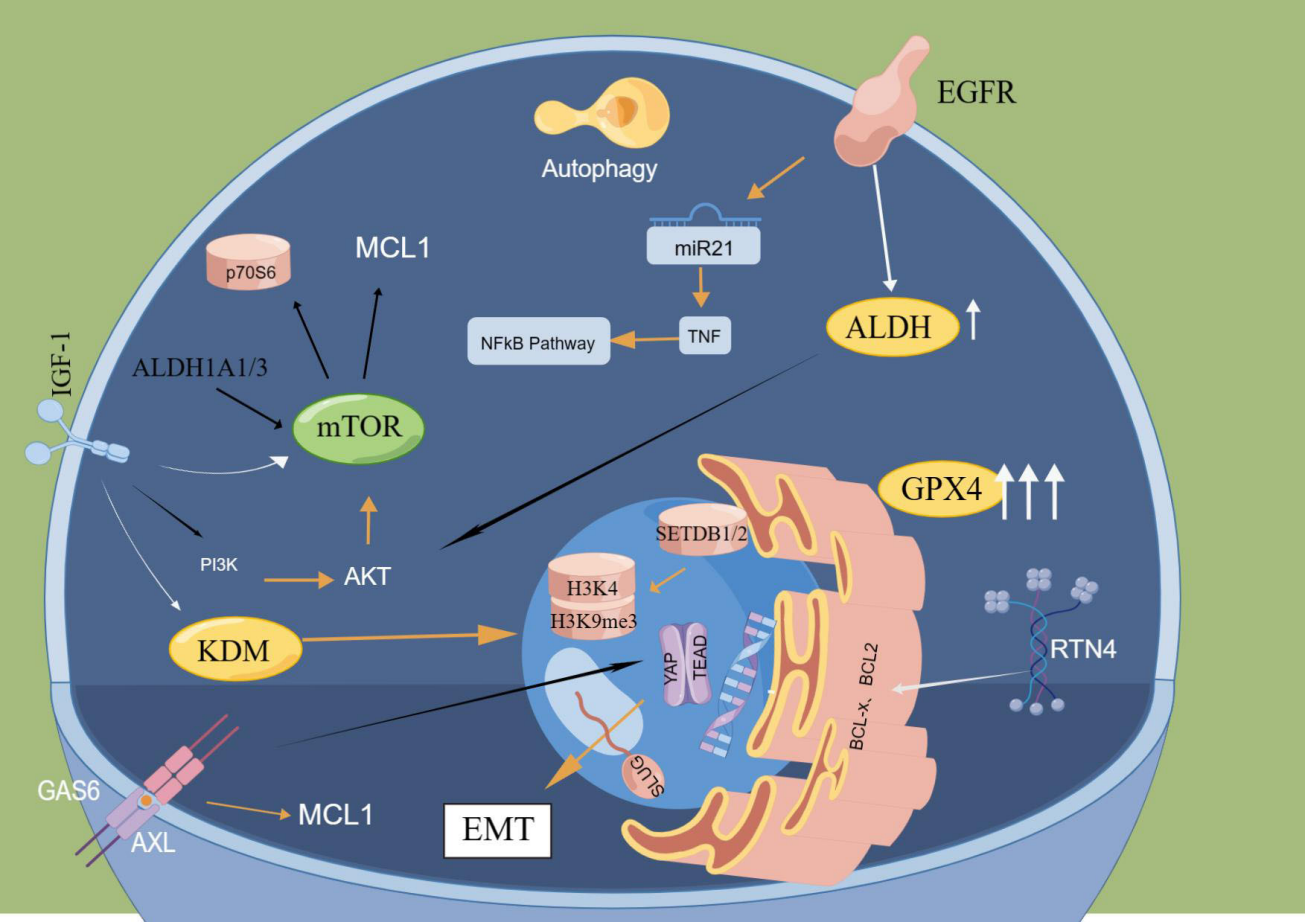
Main molecular mechanisms of DTCs. ALDH and GPX4 significantly increased in cells after drug treatment, and the formation of DTCs was notably reduced by knocking down ALDH and GPX4. In DTCs obtained from EGFr-mutated NSCLC, significant upregulation of MTOR-mediated anti-apoptotic protein MCL1 was found. Reticular protein 4(RTN4) can isolate BCL2 and BCL-x in the endoplasmic reticulum and inhibit their entry into mitochondria, thereby lowering their anti-apoptotic function. EGFR mutation can lead to downregulation of miR21 expression and activation of TNF and NFkB signaling pathways. Inhibition of IGF1R inhibited the appearance of DTP by down-regulating KDM5A and enhancing H3K4me3. The hypermethylation of H3K9me3 relates to the methyltransferases SETDB1 and SETDB2. The deletion of setDB1/2 will lead to increased expression of H3K9me3 and enhance the sensitivity of DTC to drug therapy. AXL binds to the ligand GAS6 to activate AP-tead. The EMT-related transcription factor SLUG forms a complex to inhibit apoptosis pathways.

In 2010, Sharma et al. published the first paper in *CELL* reporting DTCs, characterized by cells in a dynamically reversible state with >100-fold reduced drug sensitivity, by a mechanism whereby cells acquire drug resistance via regulating the IGF-1 signaling pathway through the histone demethylase KDM5A; this induces epigenetic changes (12). Then, several studies have identified epigenetically related KDM family members that play a vital role in the generation of DTCs and are correlated with cellular stemness and senescence (46, 47). In a variety of cell models, such as lung, breast, colorectal and melanoma models, the overall level of methylation of histone 3 lysine 4 (H3K4) was discovered to be increased and the number of DTCs was significantly reduced following the treatment of tumour cells with KDM5 inhibitors (48, 49). These studies all suggest that KDM5 functions critically in the production and maintenance of DTCs. KDM2, KDM3, KDM6 and KDM7 were all shown to be upregulated in the expression of DTCs (50, 51). In addition to altered histone methylation, DTCs also exhibit altered histone deacetylase (HDAC) activity and histone acetylation patterns, particularly elevated H3K27 acetylation and lowered H3K14 acetylation (52). Here, it should be noted that the molecular mechanism of DTC formation has attracted increasing attention from scholars and is summarized below. The molecular mechanisms of DTCs are shown in **Figure 2**.

### 3.1 DNA damage repair

DNA damage repair, which can maintain the stability of biological organisms, is achieved by homologous recombination (HR), a mechanism for DNA double-strand break repair, and DNA mismatch repair (MMR), which corrects recombination or replication errors by base exchange. However, in cancer cells, these damage repair processes are dysregulated, causing genomic instability and increased mutations. While gene mutation is closely related to tumor drug resistance. Ivana Bozic et al. (53) employed mathematical modeling to study the heterogeneity of drug resistance mutations in patients with metastatic cancer, finding that obvious mutation heterogeneity exists in all metastatic lesions, and these mutations cause resistance to drugs. By contrast, drugs can also result in genetic mutations. According to the study conducted by Luis A. Diaz et al. (54), KRAS mutations can be detected in 38% of patients with colorectal cancer who were KRAS wild-type before the treatment, after 5-6 months of treatment with panitumumab. In addition, the same phenomenon has also been found in melanoma, with additional gene mutation events found in clones after immunotherapy (55). Moreover, DNA damage repair genes are down-regulated in tumour cells as they acquire drug resistance, leading to more DNA damage (56, 57). In a colorectal cancer targeted therapy cell model, the downregulation of HR and MMR levels and increased DNA damage in DTCs can be found, causing stress mutations. The authors point out that the phenotype of DTCs can establish the optimal mutational pattern to evade drug treatment and restore proliferative activity (53, 54). This adaptive mutation has been studied in a more in-depth way in bacterial drug-resistant cells (58, 59).

### 3.2 Epigenetic alterations

With the development of single-cell sequencing technologies, it has been demonstrated that tumours are entities consisting of potentially distinct genetic and/or epigenetic subpopulations of cells. A growing number of studies have indicated that acquired drug resistance in tumours is the result of genetic mutations; besides, the same gene can have different phenotypes and tumour cells can switch between these phenotypes without genetic alterations (60), which is known as epigenetic alteration. For epigenetics, it is the key mechanism by which cells can regulate differentiation and choose their ‘cell identity’ during development (61). Recently, numerous studies have detected epigenetic changes and a vital role of epigenetics in the phenotype of DTCs.

Different from drug-resistant cells, which are genetically mutated, DTCs are characterized with more of a stage-specific, non-mutational phenotype. In early studies, it was discovered that after drug treatment of non-small cell lung cancer (NSCLC) cell populations, most cells died rapidly, with a small number of residual ‘drug-tolerant’ cells rapidly enriched, and after a ‘drug holiday’, these cells remained sensitive to the first treatment and are the same to the drug-tolerant phenotype of parental cells (12). This cannot apparently be explained by mutational mechanisms. Meanwhile, in colon cancer (62), gastric cancer (63), breast cancer (64) and melanoma (65, 66), Knoechel et al. have discovered that epigenetics plays a vital role in the development of drug-tolerant cells in leukaemia, and that the combination of epigenetic mediators can lead to better therapeutic outcomes (67). Another study showed that DTCs obtained *in vitro* from lung cancer cells treated with gefitinib had only epigenetic alterations without any genetic alterations (12). Several other studies that have reached the same conclusion manifested that tumour cells enter a drug-tolerant state through epigenetic alterations (52, 68). This non-genetic mutation mechanism promotes plasticity in DTCs. For example, when melanoma cells are treated with targeted drugs, DTCs display a neural spine stem cell state, thus favouring the generation of drug-resistant cells (66). This undoubtedly further increases the difficulty of tumour drug treatment. Furthermore, in some cell models, a small proportion of DTCs can resume normal growth and form cell populations even in the presence of drugs, indicating that in a few cases, DTCs can also mutate and cause drug resistance (12).

Epigenetic reprogramming has been found in DTCs of various tumors (69). For example, the expressions of H3K4me3 and H3K27me3 were decreased, while those of H3K9me3 were increased (70). H3K4 trimethylation is present in most promoters and is closely associated with stem cell differentiation (24). Demethylases KDM5A/JARID1A and KDM5B/JARID1B can regulate the removal of trimethylated marks on H3K4, and both of these demethylases are highly expressed in DTCs (46, 71). In melanoma, by downregulating these two demethylases, the formation of DTCs is significantly reduced, which can thus restore sensitivity to drugs (72). Studies performed by Sharma and colleagues also demonstrated that IGF1R inhibitors prevented DTCs formation by down-regulating KDM5A and enhancing H3K4me3 (12).

H3K9me3 is also the marker of cell senescence that promotes DNA methylation (52). The hypermethylation of H3K9me3 is associated with the methyltransferases SETDB1 and SETDB2. Knockout SETDB1/2 can increase the expression of H3K9me3 and enhance the sensitivity of DTC to drug therapy (39). Hp1γ, ATRX, and H3.3 mediate the formation of H3K9me3 heterochromatin, and promote the formation of DTCs (73).

### 3.3 Signalling pathways and transcriptional regulation

Taniguchi et al. discovered that in EGFR-mutated lung cancer cells, activation of the enzyme receptor AXL by blocking the negative feedback loop of SPRY4 induced the formation of DTCs (73). AXL triggers DTCs probably by binding to the ligand GAS6 and activating the GAS6/AXL axis, which can thus induce cell dormancy (74). Activation of AXL can also drive EMT, resulting in the acquisition of drug resistance (75, 76). As a transcription factor that regulates drug sensitivity, AXL is also a direct target of the YAP-TEAD complex (77, 78). Furthermore, it has also been reported that the formation of EGFR-mutated non-small cell lung cancer DTCs depends on the YAP-TEAD complex (76) that is activated to form a complex with the EMT-associated transcription factor SLUG, which inhibits the apoptotic pathway and therefore induces cells to enter a senescence-like state (45).

In response to environmental changes, cancer cells can influence signalling pathways by regulating gene and protein expression, which can thus promote cell survival and evading death (79–82). The TNFα/NFkB signalling pathway has been shown to make a vital role in tumour proliferation, metastasis and drug resistance, and in lung and breast cancer DTCs, activation of the NFkB signalling pathway has been reported and can be used as an early therapeutic target (83, 84). In EGFR-mutated lung cancer cell models, EGFR mutation leads to downregulation of miR21 expression, which stabilizes TNF mRNA and activates the TNF, thereby activating NFkB signaling pathways. In turn, activation of the NFkB signaling pathway can stimulate sustained TNF expression (85). In the same model, Hayden et al. demonstrated that EGFR inhibitors could also promote TNF receptor-associated factor 2 (TRAF2) ubiquitination and its association with RIP1/TAK1, inducing activation of the NFkB typical pathway and NFkB transcriptional targets (86). In KRAS-mutated lung cancer cells treated with EGFR inhibitors, NFkB is involved in the upregulation of type I interferons (IFNs) (87). Meanwhile, several studies have demonstrated an enrichment of IFN antiviral defence pathway signalling in EGFR-mutant lung cancer DTCs treated with targeted therapy or chemotherapy (52, 79, 79, 88). Therefore, the combined blockade of EGFR and type I IFN signalling could enhance the efficiency of EGFR inhibitors *in vitro* and result in reduced tumour cell growth *in vivo* (77).

### 3.4 Enzymes and metabolism

The metabolism of human cells and the level of protein production are adjusted based on the proliferative state of the cells (89). When DTCs enter dormancy, the corresponding metabolism decreases, depending less on glycolysis and more on mitochondrial oxidative phosphorylation (90, 91). Due to the enhanced oxidative phosphorylation of DTCs, the oxidative stress response is enhanced. Here, it should be mentioned that glutathione-dependent lipid peroxidation is an important pathway for the oxidative stress response. It has been shown that inhibition of phospholipid glutathione peroxidase 4 (GPX4) can effectively eradicate DTCs (92). Aldehyde dehydrogenase (ALDH) and NRF2 both act in the oxidative stress response of DTCs, which can thus regulate the metabolism of DTCs (93, 94). DTCs can also maintain their trophic metabolism through autophagy and fatty acid oxidation, which allows degraded macromolecules to re-enter the metabolic cycle, and facilitate mitochondrial ATP production together with acetyl coenzyme A produced by fatty acid oxidation (95, 96). In addition, the proliferation cycle of DTCs is slowed down, which protects them to some extent from damage caused by anti-tumour drugs (97).

DTCs can reduce cytotoxicity and prevent DNA damage and cell death through increasing the activity of aldehyde dehydrogenase (ALDH) and overcoming the accumulation of toxic intermediates, aldehydes (98). The use of the irreversible ALDH inhibitor disulfiram significantly reduces the proportion of DTC cells when used in combination with initial treatment (93). A study performed by Ryuhei Kawakami et al. showed that gastric cancer DTCs induced by 5-fluorouracil treatment could express various stem cell markers, including LGR5, TROY and ALDH1A3, while ALDH1A3 was more specific and stable compared to LGR5 and TROY. The number of DTCs was reduced to a great extent when ALDH1A3 was knocked down. However, the prognosis of gastric cancer patients with high ALDH1A3 expression was obviously worse than those with low ALDH1A3 expression. Mechanistically, ALDH1A3 interfered with mTOR activity and phosphorylation of the downstream p70S6 kinase, which could thus affect tumour proliferation and drug sensitivity (14). Kyung et al. also held that the mTOR pathway plays a vital role in the formation and maintenance of DTCs (24). To investigate the mechanism concerning the role of MCL-1 in the formation of DTCs, parental cells and DTCs were explored by qPCR and transcriptome sequencing, and mTOR was found to significantly regulate the expression of MCL-1 (24, 98–100). mTOR, eIF4G1, eIF4G3 and eIF3A were the four most significantly up-regulated genes in DTCs (101–103). Concerning mTOR, it inhibited the phosphorylation level of MCL-1, and knockdown of mTOR caused a significant decrease in MCL-1 expression. In addition to MCL-1, other key oncogenic proteins with short mRNA half-lives, such as are c-MYC and cyclin D1, are also regulated by mTOR-mediated translation levels, while no significant effect is observed at the RNA transcription level (104, 105).

It has also been found that DTCs rely on the GSH peroxidase GPX4 or GPX2, which can catalyze oxidative stress and prevent lipid peroxidation degrading cell membranes (92, 106). Deficiency of GPX4 causes iron death of DTC cells *in vitro* and prevents tumour recurrence *in vivo* (92). Treatment of parental cells and DTCs with the GPX4 inhibitors RSL3 and ML210, respectively, revealed massive death of DTCs with less effect on parental cells; similar results were found in a variety of tumours (8, 92, 107). A dramatic increase in 2 ‘,7 ‘ -dichlorofluorescein (DCF) staining was observed 1 h after GPX4 inhibition prior to cell death, suggesting that lipid peroxidation was present in DTCs rather than parental cells. Furthermore, the overall downregulation of Nrf2 targets and other antioxidant genes, as well as a decrease in glutathione and NADPH, weakened DTCs lipid peroxidation defenses and contributed to DTCs’ response to GPX4-dependent survival (92).

## 4 Treatment strategies for DTCs

Based on the above discussion, it can be found that lung cancer, melanoma, colorectal cancer, breast cancer and gastric cancer cells can be induced to produce a certain proportion of DTCs after drug treatment. In addition to the traditional chemotherapeutic drugs, such as oxaliplatin and fluorouracil, targeted drugs including gefitinib and oxitinib can also induce tumour cells to enter the DT state. It is shown that the DT state is characterized by the prevalence of cancer types and drug non-specificity. Cancer cells isolated from cell lines with a monoclonal clone could also form DTCs under drug treatment and in almost the same proportion as the parental cells (12). This implies that the DT state is an intrinsic property of the cancer cells themselves, rather than a characteristic of some individual cancer cells. Therefore, it is demonstrated that the DT state is an intrinsic property of cancer cells and not a characteristic of some individual cancer cells. Therefore, the study on the DT state is of general value for cancer therapy.

In summary, the discovery of the DT state provides a new therapeutic opportunity for cancer treatment. Targeting DT cells for treatment at this stage not only improves the effectiveness of cancer drug therapy and directly reduces the probability of cancer recurrence, but also has the potential to improve the chances of curing cancer by lowering or even eliminating the production of stable drug-resistant cells.

As the mechanisms of DTC production and maintenance are still not fully understood and involve the regulation of multiple proteins and signalling pathways, combination therapy with multiple drugs remains the primary strategy for the treatment of tumour resistance (108, 109). The combination of multiple drugs remains the primary strategy for treating tumour resistance. Based on the research on epigenetics in DTCs, it has been demonstrated that targeting epigenetic modifiers, including the lysine demethylase KDM5, significantly reduce the number of DTCs in cellular models of melanoma, colon and breast cancer (46). Targeting the tumour microenvironment may also be a valuable therapeutic strategy. For example, BRAF mutant melanoma cells initially respond to BRAF inhibition but rapidly show resistance to the drug when in close contact with stromal cells. From a mechanistical perspective, melanoma-associated fibroblasts respond to BRAFi by enhancing the production of fibronectin, which enhances melanoma cell focal adhesion kinase (FAK) signalling, and co-suppression of BRAF and FAK prevents reactivation of mitogen-activated protein kinases (MAPK), causing more effective control of tumour growth (110). Alterations in the metabolic and transcriptional processes of DTCs may also serve as therapeutic targets. MAPK inhibition induces an RXRG-driven stem-like transcriptional program in melanoma cells, and RXR antagonism reduces the number of DTCs and delays drug resistance in tumour cells (63).

DTCs are theoretically dormant for an extended period of time, giving the immune system and drugs a window of time to clear DTCs. For instance, in mouse models of lung and pancreatic cancer, drug-induced cancer senescence and SASP activation have been demonstrated to enhance immune cell activity and thus clear tumour cells (111, 112). Several studies have identified that epigenetically related KDM family members (KDM2, KDM3, KDM5, KDM6, KDM7) play a significant role in generating DTCs. Besides, it was found that KDM expression was upregulated in various cell models including lung, breast, colorectal and melanoma ones, and treatment of tumour cells with KDM inhibitors resulted in increased overall levels of histone methylation and a significant reduction in the number of DTCs. Here, it should be noticed that KDM inhibitors may be a new approach to the treatment of DTCs.

The dependence of cancer cells on oncogenic signalling stress response pathways is closely related to oncogenicity, and pharmacological interference with oncogenic signalling can enhance the effectiveness of therapeutic stress pathways. Therefore, blocking both oncogenic signalling and stress response pathways is considered to be the best treatment for DTCs (42). For example, cytokine stimulation can reverse the drug resistance of AML stem cells and make them sensitive to chemotherapy (113). Due to the dynamic nature of the drug-resistant phenotype of DTCs, the timing of targeted therapy may also be a critical factor in treatment outcome (40).

## 5 Outlook

DTCs are of great importance in tumour-acquired drug resistance. Although there has been a great deal of research on DTCs, their origin, markers and specific molecular mechanisms still remain unclear. Therefore, no effective therapeutic strategies targeting DTCs were proposed. In more studies, it has been concluded that DTCs occur mainly at a non-genetic epigenetic level, and therefore whole-transcriptome sequencing may not be an appropriate approach (40). The study of whole-transcriptome sequencing may not be appropriate, but should focus more on proteomic alterations. A cluster of DTCs may generate different drug-resistant clones, thus increasing the difficulty of treatment (8). Fortunately, one of the characteristics of DTCs is that they remain dormant for a longer period of time, giving us the opportunity to treat them. Therefore, searching for specific markers and efficient protein signalling pathways to effectively target them may be the future direction of research on DTCs.

## Author contributions

X-WL and B-L: Paper writing and data collection; J-CC, ZC, F-rC and XL: data collection; S-ZW and J-CW: Supervision and paper revision. All authors contributed to the article and approved the submitted version.
